# What is the difference in TOS progression secondary to complete vs. incomplete cervical ribs?

**DOI:** 10.1002/ccr3.4311

**Published:** 2021-06-10

**Authors:** Ammara Bint I Bilal, Fateen Ata, Mohamed A. Yassin

**Affiliations:** ^1^ Department of Radiology Hamad Medical Corporation Doha Qatar; ^2^ Department of Internal Medicine Hamad Medical Corporation Doha Qatar; ^3^ Department of Medical Oncology/Hematology National Centre for Cancer Care and Research Hamad Medical Corporation Doha Qatar

**Keywords:** bilateral cervical ribs, neurovascular TOS, thoracic outlet syndrome

## Abstract

When complete cervical ribs are present, clinicians and surgeons should look out for the possibility of arterial involvement with disease progression similar to what happened in this case.

## Graphical abstract

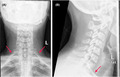

## 1 CLINICAL IMAGE CASE

We present an X‐ray‐cervical spine showing bilateral complete cervical ribs of a young female patient presenting with progressively worsening neurovascular thoracic outlet syndrome. She was managed conservatively with physical therapy, pain management, and has a high probability of surgical intervention.

A 36‐year‐old, previously healthy female patient was referred with chronic agonizing neck pain and bilateral hand numbness with variable grip strength being proportional to the amount of exertion during her work as a teacher. Physical examination denoted tenderness at the mid‐cervical spine, decreased power of both biceps, and intact distal pulses bilaterally.

**FIGURE 1:**
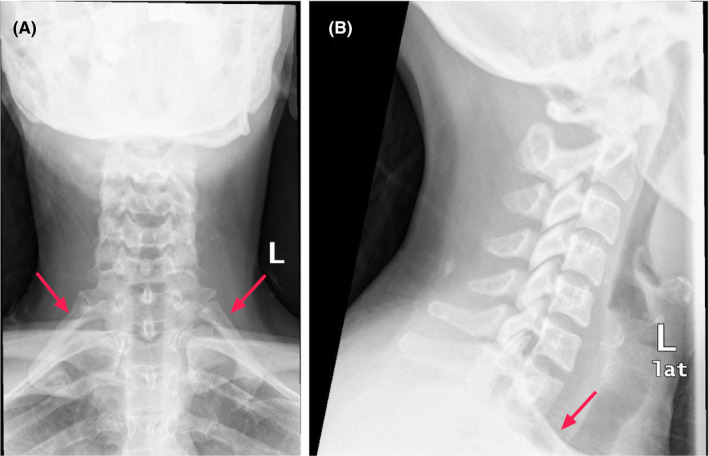
Cervical X‐ray showing bilateral complete cervical ribs (red arrows) Left: AP view, Right: Lateral view

X‐ray for the cervical spine was ordered, which revealed bilateral complete cervical ribs [Figure 1]. After involving neurology and thoracic surgery, a conservative management route was adopted. Over the subsequent 2 years, her neurogenic symptoms aggravated, and vascular manifestations appeared asymmetrically, pain worsened, and she developed atrophy of the left thenar and hypothenar muscles. Partial compression of the left subclavian artery and vein eventually developed as per CT scan. Vascular surgery discussed surgical intervention, and the patient awaits surgery.

Cervical ribs, a rare anatomic variant, are supernumerary ribs arising from the 7th cervical vertebra.^1^ With a prevalence of 1.1% among the healthy population, they can be complete or incomplete and have a 25% higher incidence in patients with thoracic outlet syndrome.^1^ Thoracic outlet syndrome results from neural and/or vascular compression of structures passing through the interscalene triangle; the presence of cervical ribs effectively constricts this pathway's boundaries.^1^ It has been investigated that almost all arterial TOS secondary to cervical ribs are associated with complete cervical ribs rather than incomplete.^2^ Treatment is challenging, and when complete cervical ribs are present, clinicians and surgeons should look out for the possibility of arterial involvement with disease progression similar to what happened in this case.

## CONFLICT OF INTEREST

None declared.

## AUTHOR CONTRIBUTIONS

AB: involved in literature review, manuscript writing, and image selection. FA: involved in literature review and revisions in the manuscript. MAY: involved in literature review, supervision, and revisions in the manuscript.

## ETHICAL APPROVAL

Written consent was taken from the patient for the case and accompanying images before submission. The case was approved by the Medical Research Centre (MRC), Qatar, before submission.
